# Preparation of CeO_2_/UiO-66-NH_2_ Heterojunction and Study on a Photocatalytic Degradation Mechanism

**DOI:** 10.3390/ma15072564

**Published:** 2022-03-31

**Authors:** Ziwei Liu, Yanli Zhuang, Limin Dong, Hongxu Mu, Shuo Tian, Leiming Wang, Aoxiang Huang

**Affiliations:** Heilongjiang Provincial Key Laboratory of CO_2_ Resource Utilization and Energy Catalytic Materials, School of Material Science and Chemical Engineering, Harbin University of Science and Technology, Harbin 150040, China; liuziwei199604@163.com (Z.L.); hrbust_mhx@163.com (H.M.); tianshuo199606@163.com (S.T.); wangleiming199905@163.com (L.W.); hover1002@163.com (A.H.)

**Keywords:** CeO_2_, UiO-66-NH_2_, heterojunction, photocatalytic mechanism

## Abstract

CeO_2_/UiO-66-NH_2_ (marked as Ce/UN) composites were in-situ synthesized by a hydrothermal method. The properties, photocatalytic aspects, and degradation mechanism of Ce/UN were studied carefully. SEM results show that Ce/UN have a 3D flower-like structure, where octahedral UiO-66-NH_2_ nanoparticles are embedded in the two-dimensional sheet of CeO_2_. TEM results demonstrate that CeO_2_ and UiO-66-NH_2_ are bonded interfacially to constitute a hetero-junction construction. Data obtained by electrochemical impedance spectroscopy and fluorescence spectroscopy established that Ce/UN has less charge shift resistance and luminescence intensity than these of two pure substances. When the ratio of Ce/UN is 1:1, and the calcination temperature 400 °C is used, the degradation efficiency of RhB in photocatalysis by obtained Ce/UN is about 96%, which is much higher than in the case of CeO_2_ (4.5%) and UiO-66-NH_2_ (54%). The improved photocatalytic properties of Ce/UN may be due to the formation of hetero-junction, which is conducive for most photo-carriers and thus the interfacial charge shift efficiency is enhanced. By the free radical capture test, it can be inferred that the major active substances involved in the degradation related to photocatalysis is H^+^ and $\cdot \;O_{2}^{-}$.

## 1. Introduction

Fast industry development in the last few decades leads to increased environmental pollution, which has to be solved, whereas renewable sources and environmental problems have attracted a lot of attention by the researchers [1,2,3,4]. The research on environment-friendly and sustainable energy composite materials will become an important research direction in the future. Rare earth oxides have the advantages of low price, stability and non-toxicity and in the light catalysis, batteries, and other fields have a wide range of applications [5,6]. Cerium oxide (CeO_2_) has good conductivity and stability, and has high oxygen storage capacity because of the existence of reversible REDOX pairs of Ce^3+^/Ce^4+^, which enables it to have strong electronic interaction with other components at the interface [7]. Therefore, it has potential application as a carrier material in catalytic oxidation reactions. However, cerium oxide has a wide band gap and limited absorption of visible light, and its photoelectron generation and hole recombination rates are high, leading to its poor photocatalytic degradation performance [8,9]. Numerous researchers have stated that photocatalytic properties of materials can be improved by improving morphology, crystal plane regulation, substance doping, or heterojunction construction [7,10]. For example, Wu and Shen [11,12] prepared CeO_2_/Co_3_O_4_ composite materials by a biological template method and solvothermal method, and improved the electron transmit rate of the materials by regulating the morphology of the composite materials. Pu and Chae [13,14] prepared Cu_2_O/CeO_2_ composite materials by calcination and sol-gel electrostatic spinning. By constructing p-n junction, the absorption of CeO_2_ under visible light and separation efficiency of a light carrier were improved. Other researchers prepared CdS/CeO_2_ composites by two-step hydrothermal method, one-pot method, and solvothermal method, and improved the photocatalytic performance of the composites by constructing appropriate band gap matching [15,16,17].

Metal-organic framework compounds (MOFs), as a new type of substance different from semiconductors, have great potential in photocatalytic reactions because of their high specific regions on the surface, flexible porous size and design of internal construction [18,19,20]. As a photocatalytic agent, it can provide an additional pathway for photoelectron migration, thereby boosting the segregation of electrons and holes (e^−^–h^+^), in turn, improving the efficiency of composites in terms of photocatalysis. However, poor electrical conductivity, intrinsic catalytic activity, and stability affect its application in catalytic reactions. Researchers synthesized UiO-66-NH_2_ MOFs with BiOBr [21,22], Ag_3_PO_4_ [23], CdS [24,25,26], and other semiconductor materials to structure a composite. The results show that the heterojunction structure is formed at the interface between MOFs and semiconductor substances, which can effectively inhibit the rapid restructuring of e^−^–h^+^, thus improving the properties of semiconductor substance in terms of photocatalysis [27,28]. 

Considering the low efficiency in photocatalysis, poor adsorption capacity, and low utilization rate of visible light of CeO_2_, this paper intends to promote the separation efficiency of e^−^–h^+^ by constructing heterojunction. Meanwhile, the big specific surface areas of MOFs is used to improve the adsorption capacity of pure substances, and finally realize the improvement of the photocatalytic degradation performance of the material. Therefore, Ce/UN composite photocatalytic material was in-situ synthesized by a hydrothermal method in this paper, mainly studying the influence of preparation process and composite ratio on the photocatalytic degradation performance of composite materials, and revealing the photocatalytic degradation mechanism.

## 2. Materials and Experimental Procedures

All chemicals and reagents used in this paper are analytical grade reagents, bought from Shanghai Maclean Biochemical Technology Co., Ltd. (Shanghai, China), and have not been more purified.

### 2.1. Preparation of CeO_2_ Nanosheets

**Figure 1:**
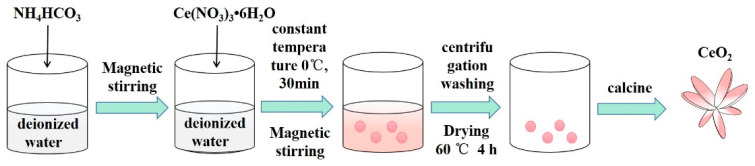
Diagrammatic sketch of the synthesis of CeO_2_ nanosheets.

Furthermore, 1.39 g Ce(NO_3_)_3_•6H_2_O and 0.75 g NH_4_HCO_3_ were separately dissolved in 200 mL deionized water. Then, the NH_4_HCO_3_ liquor was quickly poured into the Ce(NO_3_)_3_•6H_2_O solution and kept at 0 °C for 30 min. The mixed solution was filtered through a funnel to collect the products and washed with deionized water 3 times. The collected precipitate was dried at 69 °C for 4 h to get the precursor of CeO_2_. Finally, the precursor of CeO_2_ was calcined at 350 °C, 400 °C and 450 °C for 4 h to obtain CeO_2_ nanosheets. The preparation process of CeO_2_ nanosheets is according to Figure 1. 

### 2.2. Preparation of Ce/UN Composites

**Figure 2:**
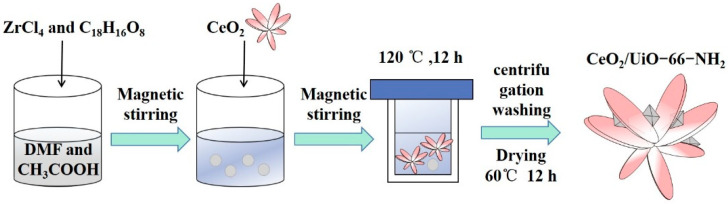
Diagrammatic sketch of the synthesis of Ce/UN composites.

Ultrasonic dispersion of 20.4 mg ZrCl4 and 14.5 mg 2-amino-1, 4-carboxylic acid in 10 mL dimethylformamide and 1.2 mL acetic acid mixed solvent, the prepared CeO_2_ nanosheets were dissolved in the mixed solvent, and then the mixed solution was loaded into the teflon lined hydrothermal autoclave. It was kept warm for 12 h at 120 °C, and then centrifuged it three times with deionized water. The lamellar Ce/UN composite photocatalytic material was prepared by drying for 12 h at 60 °C. The preparation process of Ce/UN composites is referring to Figure 2. Synthetic quality ratio of 0.5:1, 1:1, 1.5:1, and 2:1 Ce/UN substance labels were 0.5-Ce/UN, 1-Ce/UN,1.5-Ce/UN, and 2-Ce/UN.

### 2.3. Properties Characterization

The phase of the substance was characterized by a Model D/MAX-3B X-ray diffractometer (λ = 0.154 nm) manufactured by Tokyo Company, Japan. The diffraction target of the instrument was Cu-Ka (scanning rate was 4°/min), and the scanning Angle 2θ ranged from 10° to 80°. FEI Sirion200 (scanning electron microscopy, SEM, FEI Company, Hillsboro, FL, USA) was employed in looking into the microstructure and size of the material. The crystal structure and lattice spacing of photocatalytic substances were featured by transmission electron microscopy (TEM, JEM2100, JEOL, Showa, Japan). X-ray photoelectron spectroscopy (XPS, Thermo Escalab 250Xi, Thermo Fisher, Waltham, MA, USA) was used to determine the composition of the material. The surface area of the catalyst and the pore size distribution was measured using a Brunauer–Emmett–Teller surface area analyzer (BET, Micromeritics ASAP 2460, Micromeritics Instrument Corp, Shanghai, China). Fluorescence spectrophotometer (Shimadzu RF-5301, PL, Shimadzu Corporation of Japan, kyoto, Japan) was used to analyze the photoelectron–hole recombination rate of the samples. The excitation wavelength was 325 nm. The photocatalytic effect of the materials was measured by uV-757 CRT, and BaSO_4_ was used as reflection reference substance.

### 2.4. Photocatalytic Performance Test

The photocatalytic effect of simulated organic dye wastewater (20 mg/L rhodamine B (RhB)) was evaluated at room temperature. A 300 W xenon lamp (GX2500, Shanghai Jiguang Special Lighting Electric Appliance Factory, Shanghai, China) was used as a simulated solar light to test the light catalytic performance of the prepared samples. Take 50 mL RhB into a beaker and add 50 mg photocatalytic material. Firstly, the beaker was stirred for 1 h under no light to achieve the dynamic equilibrium of adsorption–desorption on the surface of the composite material. Then, 6 mL of the mixed solution was placed in a centrifugal tube. After 10 min of centrifugation, the supernatant was taken and placed in a quartz colorimetric dish. The initial absorbance was measured and marked as A0. Turn on the simulated light for irradiation, then take out 4 mL of the mixed solution every 30 min and repeat the above steps. Take the supernatant and place it in a quartz coloration to measure the absorbance of the dye (A1, A2, A3···).

### 2.5. Electrochemical Measurement

Electrochemical impedance spectroscopy (EIS) was carried out on a VMP3 workstation, as was Mott–Schottky. The workstation is equipped with three electrodes, including glassy carbon, platinum, and saturated calomel. The preparation method of the working electrode was as follows: 2 mg sample was placed in a small sample tube, 200 μL ethanol reagent was added, and ultrasonic dispersion for 1 h. The above sample suspension is moved with a suction gun and laid on the conductive layer of the glassy carbon electrode, and it can be dried. During the experiment, Na_2_SO_4_ (0.5 M) was used as electrolyte solution. EIS was tested in the range of 0.01 kHz to1 kHz. Motshottky (MS) plots were measured at 1 kHz and the voltage was −1–1 v.

## 3. Results and Discussion

### 3.1. XRD Analysis

**Figure 3:**
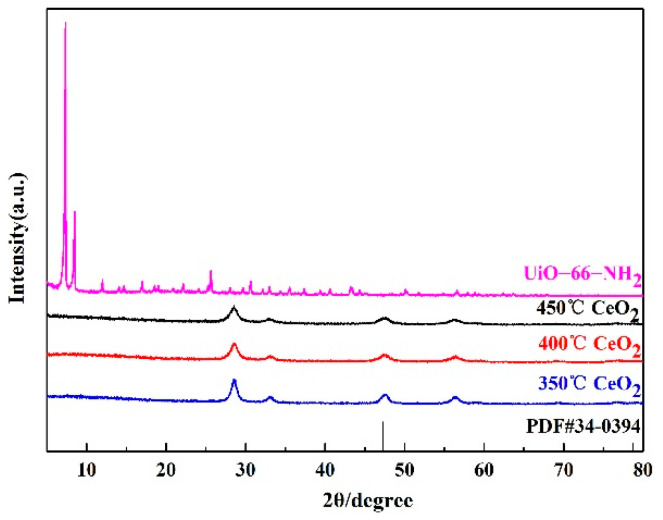
XRD patterns of CeO_2_ prepared at different temperatures and UiO-66-NH_2_.

**Figure 4:**
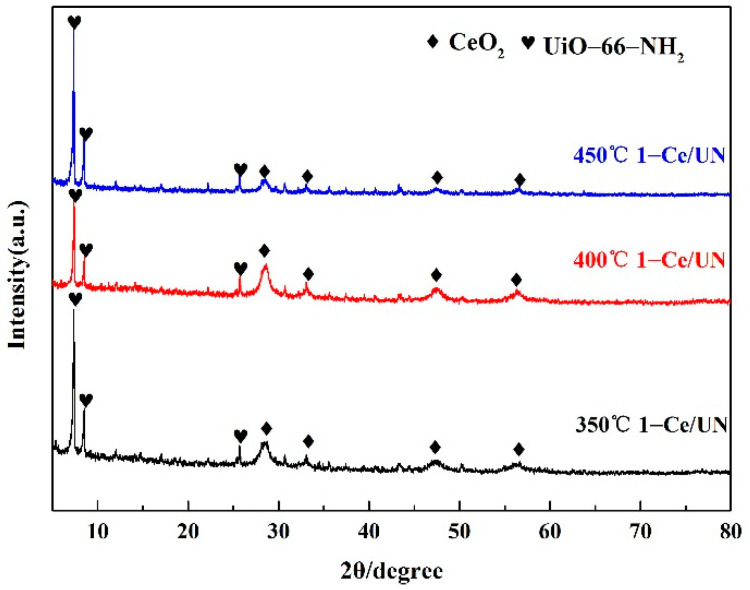
XRD patterns of 1-Ce/UN at different temperatures.

**Figure 5:**
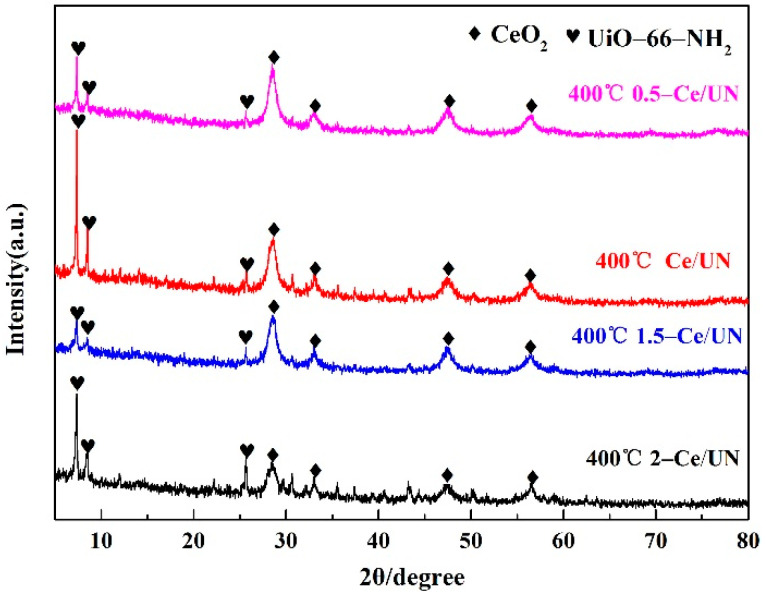
XRD patterns of 400 °C Ce/UN with different composite proportions.

Figure 3 illustrates the X-ray diffraction patterns for the CeO_2_ prepared at different temperatures and UiO-66-NH_2_ substance. It can be observed that the diffraction peak of the CeO_2_ samples prepared at different temperatures are consistent with the standard card (JCPDS 34-0394) peak. The characteristic peaks at 2θ = 28.55°, 33.08°, 47.48°, 56.33°, 59.09°, 69.4°, 76.7°, and 79.07° correspond to (111), (200), (220), (311), (222), (400), (311) and (420) of cubic phase CeO_2_, respectively. This indicates that CeO_2_ synthesized in this paper is a cube structure and its space group is Fm-3m (225). Moreover, it can also be displayed from Figure 3 that the X-ray diffraction peaks of the UiO-66-NH_2_ made are the same as those of literature and simulation [29,30], explaining that the UiO-66-NH_2_ material has been synthesized in this paper. Figure 4 states the XRD peaks of 1-Ce/UN at different temperatures. It can be observed that the characteristic peak of cubic CeO_2_ is the strongest at 400 °C with the increase of temperature, and the phase crystallization is the best at this temperature. Figure 5 is XRD patterns of 400 °C Ce/UN with different composite proportions. Referring to Figure 4 and Figure 5, the XRD peaks of Ce/UN with diverse proportions and different temperatures are consistent with the representative peaks of UiO-66-NH_2_ and CeO_2_. No other XRD peaks were found due to any impurities, indicating the high purity of the composite. The major diffraction peaks of the composites at 7.3° and 8.4° correspond to the typical peak of UiO-66-NH_2_, and the pattern is maintained well. The diffraction reflections at 28.55°, 33.08°, 47.48°, and 56.33° are related to (111), (200), (220), and (311) crystal planes of CeO_2_, which clearly indicates the phase formation of Ce/UN nanocomposites. The strong and sharp diffraction peaks indicate that Ce/UN nanocomposites have good crystallinity.

### 3.2. SEM Analysis

**Figure 6:**
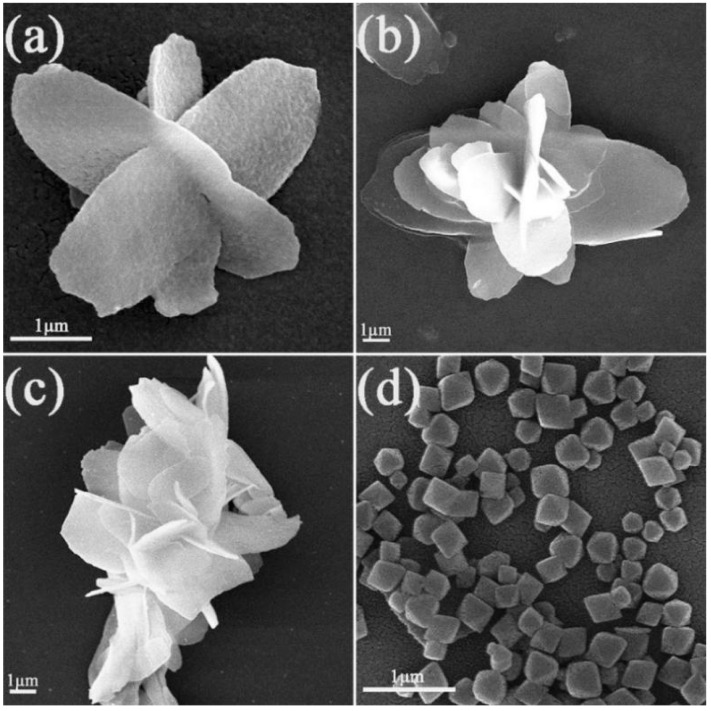
SEM images of (**a**) 350 °C CeO_2_, (**b**) 400 °C CeO_2_, (**c**) 450 °C CeO_2_, and (**d**) UiO-66-NH_2_.

The microtopography of CeO_2_ calcined at different temperatures and UiO-66-NH_2_ were analyzed by SEM, as shown in Figure 6. According to Figure 6a–c, the CeO_2_ calcined at different temperatures all present a 3D flower-like structure. The length of CeO_2_ sheet is about 5 μm, and the width is about 2 μm (as shown in Figure 6a). In addition, the nanosheets gradually increase in size with increasing temperature, which provides a large contact area for the transfer of e^−^−h^+^, and can be loaded on UiO-66-NH_2_ well. From Figure 6d, it can be observed that the UiO-66-NH_2_ is a particle with a regular octahedral structure. Its average size is about 200–500 nm.

**Figure 7:**
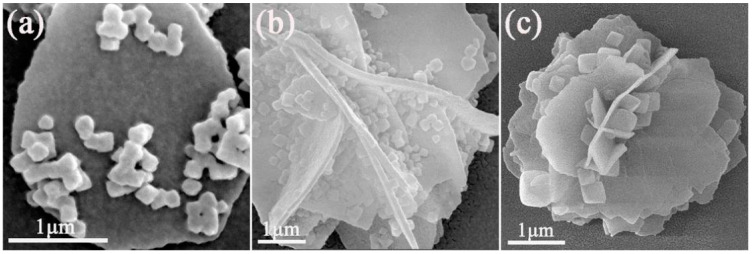
SEM morphologies of Ce/UN composites: (**a**) 350 °C 1-Ce/UN; (**b**) 400 °C 1-Ce/UN; (**c**) 450 °C 1-Ce/UN.

**Figure 8:**
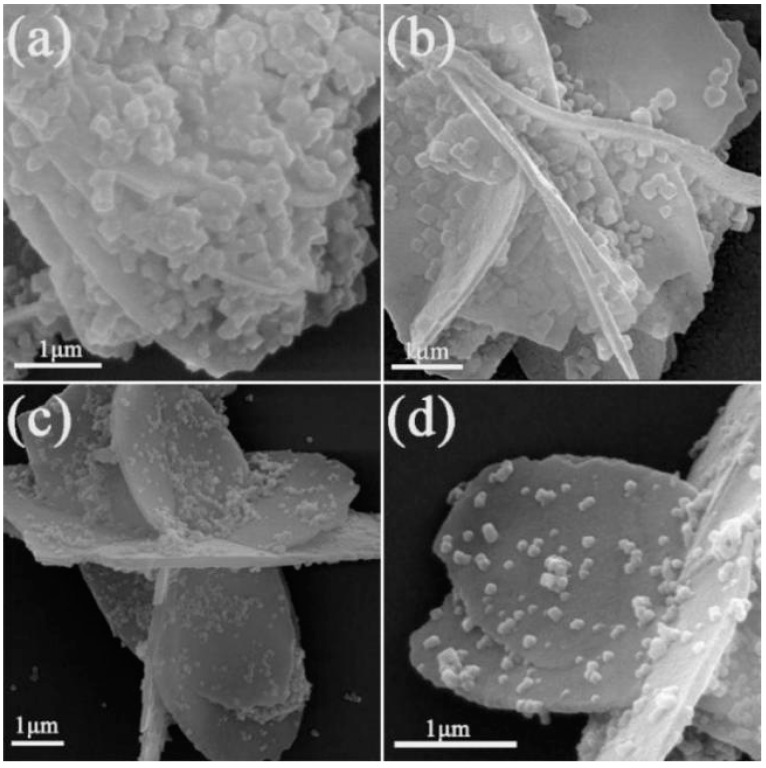
SEM morphologies of Ce/UN composites with different proportions: (**a**) 0.5:1; (**b**) 1:1; (**c**) 1.5:1; (**d**) 2:1.

**Figure 9:**
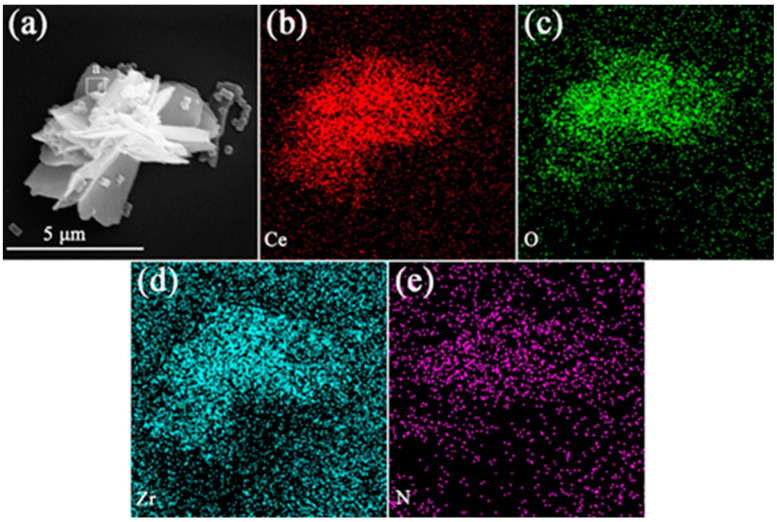
Elements maps analysis of 400 °C 1-Ce/UN composites: (**a**) SEM image; (**b**–**e**) element analysis of Ce, O, Zr and N.

Figure 7 shows the microstructure of CeO_2_ calcined at different temperatures and UiO-66-NH_2_ composites in the 1:1 ratio. It is not difficult to find that particles of UiO-66-NH_2_ are covered on pure CeO_2_ nanosheets, and the diameter of UiO-66-NH_2_ is about 200–500 nm. During sample preparation, ultrasonic treatment does not separate UiO-66-NH_2_ from CeO_2_, instructing that UiO-66-NH_2_ is well attached to CeO_2_. The interface formed between ions may have good interaction. Figure 8 shows the morphologies of CeO_2_ (calcined at 400 °C) and UiO-66-NH_2_ composites in different proportions. It can be observed that the agglomeration of UiO-66-NH_2_ nanoparticles on CeO_2_ flower nanosheets decreases with the decrease of the content of UiO-66-NH_2_. When the ratio of CeO_2_ to UiO-66-NH_2_ is 1:1, the Ce/UN composites have the best photocatalytic properties of all substances (This will be studied in detail in the photocatalytic experimental section), so the focus of detailed study is on 400 °C 1-Ce/UN. Figure 9 presents the element mapping analysis on the 400 °C 1-Ce/UN composites. It can be seen that the elements of Ce, O (specific elements of CeO_2_), Zr and N (representative element of UiO-66-NH_2_) appear in the Ce/UN composites and are evenly distributed. Table 1 shows the SEM-EDS analysis of area “a” signed in Figure 9a. The results show that the analyzed region may contain CeO_2_ and UiO-66-NH_2_. Combined with the XRD test results (Figure 4 and Figure 5), it can be further inferred that the Ce/UN composite materials were synthesized.

### 3.3. TEM Analysis

**Figure 10:**
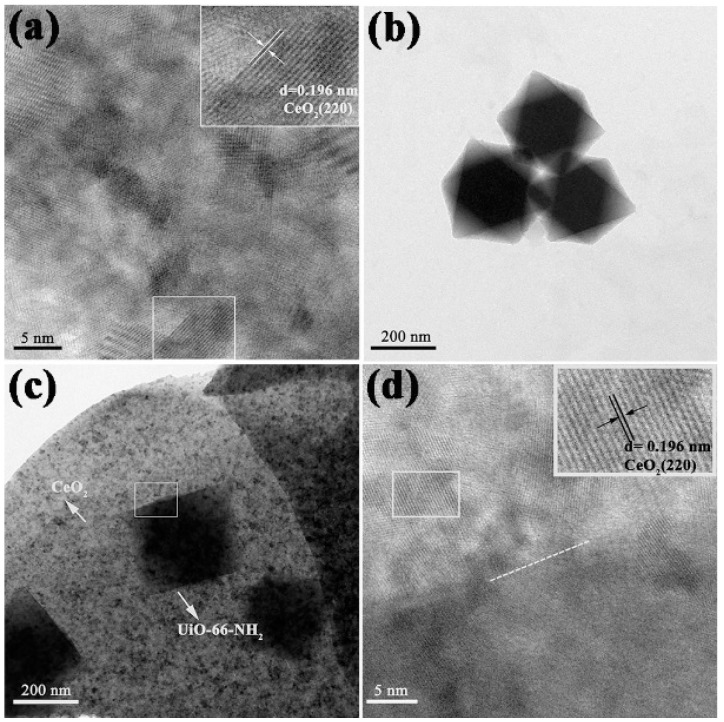
TEM and HRTEM images of samples: (**a**) CeO_2_; (**b**) UiO-66-NH_2_; (**c**) 400 °C 1-Ce/UN composites; (**d**) magnified HRTEM image of the white box labelled in (**c**).

The shape and interfacial structures of CeO_2_, UiO-66-NH_2_, and 400 °C 1-Ce/UN were analyzed by TEM and HRTEM, as shown in Figure 10. According to Figure 10a, the lattice fringe spacing of CeO_2_ is about 0.196 nm, which is well matched with the (220) crystal plane. TEM images shown in Figure 10b indicate that UiO-66-NH_2_ has an octahedral construction with a maximum length of about 300 nm. Figure 10c further demonstrates that UiO-66-NH_2_ is finely scattered on the flower-like structure of CeO_2_. Octahedral UiO-66-NH_2_ with a surface diameter of about 300 nm is in close contact with flower-like CeO_2_, which matches the XRD results. In addition, according to the Figure 10d, the interface between CeO_2_ and UiO-66-NH_2_ can be clearly observed (shown as the white dotted line in Figure 10d), confirming the composition of heterostructures between them. These results suggest that UiO-66-NH_2_ was successfully grown on CeO_2_ flower nanosheets and interlinked, which may contribute to the segregation of photogenerated carriers.

### 3.4. XPS Analysis

**Figure 11:**
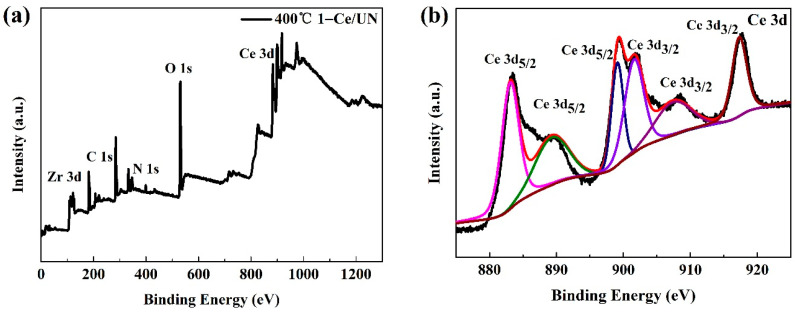

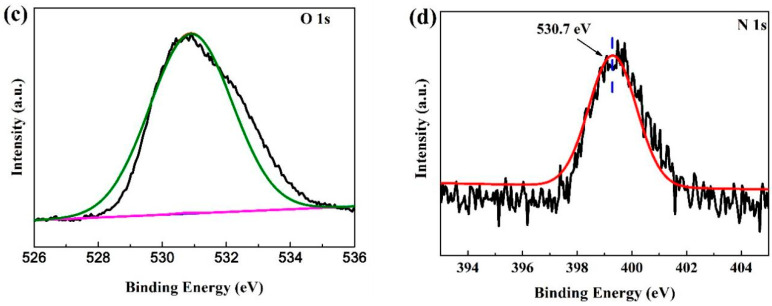
XPS spectra of 400 °C 1-Ce/UN catalyst: (**a**) Survey, (**b**) Ce3d, (**c**) O1s, (**d**) N1s.

The chemical states and bonding environment of 400 °C 1-Ce/UN were studied by XPS analysis. The results are shown in Figure 11. The total measurement spectrum (Figure 11a) confirmed that Ce/UN heterostructure composite contained Ce, O, Zr, and N elements, which was consistent with the element distribution diagram of SEM-EDS (Figure 9). For the high-resolution spectrum of Ce 3d, the 883.4 eV, 889.6 eV, and 899.3 eV characteristic peaks shown in Figure 11b are attributed to the binding energy of Ce 3d_5/2_. In addition, these three peak-to-peak values are 901.9 eV, 907.8 eV, and 917.6 eV, respectively, which belong to the binding energy of Ce 3d_1/2_ [31,32]. In Figure 11c, the binding energies of Ze 3d_5/2_ and Zr 3d_3/2_ are about 182.4 eV and 184.7 eV, respectively [33]. This demonstrates the existence of Zr^4+^ in zirconium oxygen clusters. In addition, the O1s peak at 529.7 eV in Figure 11d belongs to the lattice oxygen in Ce-O bond and Zr-O bond [4,34].

### 3.5. BET Analysis

**Figure 12:**
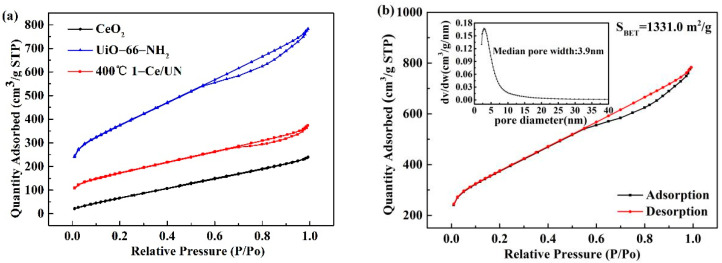

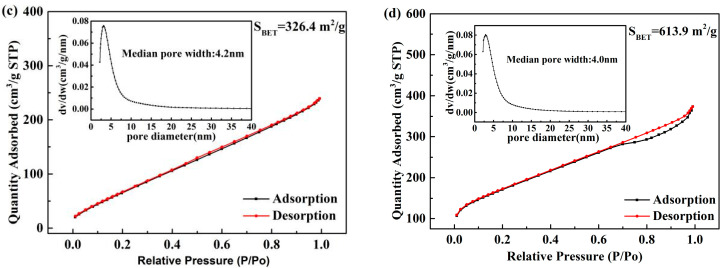
(**a**) N_2_ adsorption–desorption isotherms of CeO_2_, UiO-66-NH_2_ and 400 °C 1-Ce/UN; (**b**) N_2_ adsorption–desorption isotherms and the pore size distribution of UiO-66-NH_2_; (**c**) N_2_ adsorption–desorption isotherms and the pore size distribution of CeO_2_; (**d**) N_2_ adsorption–desorption isotherms and the pore size distribution of 400 °C 1-Ce/UN.

Figure 12a displays that the N_2_ adsorption–desorption isotherm was applied to investigate the specific surface area and pore size distribution of synthetic materials. It could be found that the specific surface area of 400 °C 1-Ce/UN was higher than that of CeO_2_ after introducing UiO-66-NH_2_, indicating that UiO-66-NH_2_ could significantly increase the specific surface area of the composites. As shown in Figure 12b, the values of BET surface area of UiO-66-NH_2_ are 1331.0 m^2^/g, and UiO-66-NH_2_ displayed a typical type IV isotherm with a H3-shaped hysteresis loop observed in the range 0.6–1.0 P/P_0_, indicating its mesoporous structure based on the IUPAC classification. In addition, the pore size distribution confirmed that the pores in UiO-66-NH_2_ were 3.9 nm, which mainly distributed in the range 2–50 nm, corresponding to a mesoporous structure. According to Figure 12c, the specific surface areas of CeO_2_ were 326.4 m^2^/g and the pore size was 4.2 nm. The pore size distribution results showed that CeO_2_ is mesoporous. Among the composites, the BET specific surface area of 400 °C 1-Ce/UN is 613.9 m^2^/g, and the pore size is 4.0 nm (as shown in Figure 12d). Although the specific surface area of 400 °C 1-Ce/UN is lower than that of UiO-66-NH_2_, it is much higher than CeO_2_, demonstrating that 400 °C 1-Ce/UN heterojunction can provide abundant surface active sites and facilitate photoexcited charge carriers transfer [35].

### 3.6. Optical Properties

**Figure 13:**
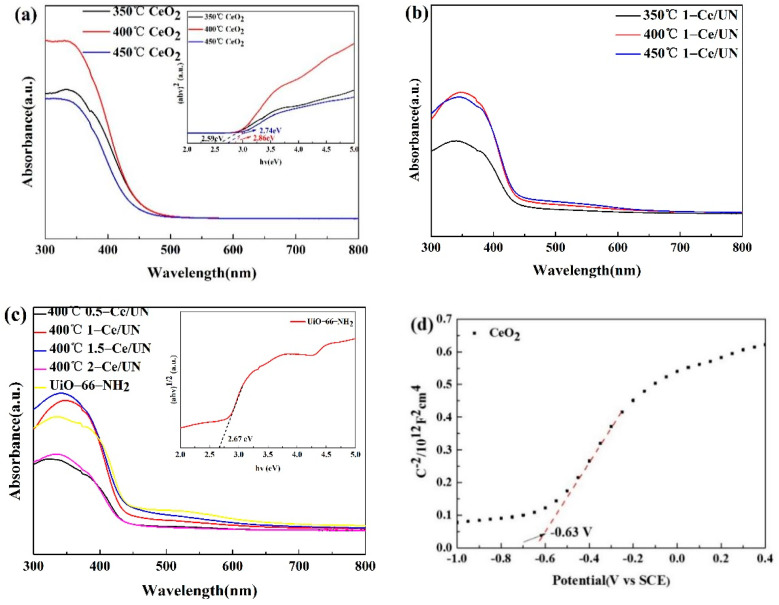

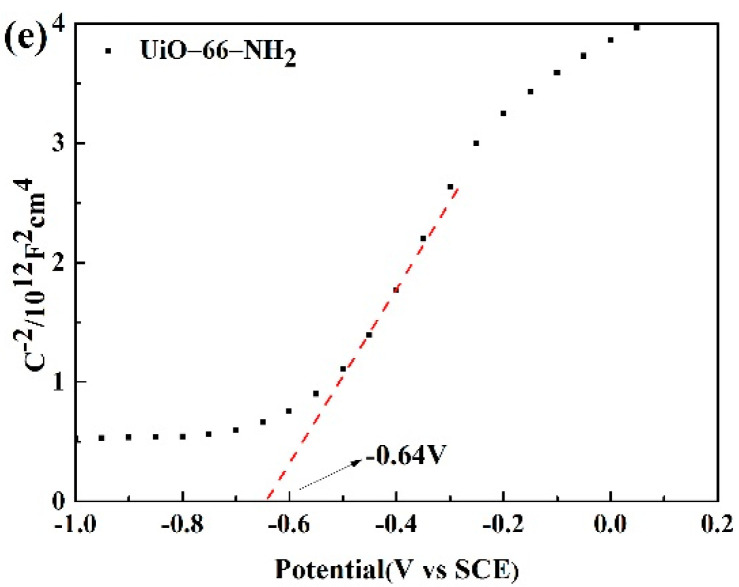
UV-Vis reflectance spectrum of (**a**) CeO_2_ prepared at different temperatures, (**b**) (350 °C, 400 °C, 450 °C)1-Ce/UN, (**c**) Ce/UN composites with different composite ratios, and the Mott–Schottky curve of (**d**) CeO_2_ and (**e**) UiO-66-NH_2_.

The characteristics of absorption in optics and band gap of CeO_2_, UiO-66-NH_2_ and Ce/UN were studied by using UV-vis DRS, and then the visible light utilization rate of the composites was analyzed, as shown in Figure 13. According to Figure 13a, the light absorption values of CeO_2_ are about 430 nm, respectively, which are in good agreement with the results of previous studies [36,37]. In addition, according to Figure 13a,b, compared with CeO_2_, 400 °C 1-Ce/UN showed a longer wavelength absorption edge. In addition, the edge of the absorption peak is about 440 nm. Therefore, introducing UiO-66-NH_2_ into CeO_2_ can boost its own light utilization property, and the Ce/UN heterojunction makes the absorption edge of the material move to the right, resulting in a larger absorption wavelength, so that it can absorb more visible light.

The visible light absorption performance of Ce/UN heterojunction shows that it can be a good visible- light absorption semiconductor material. Typically, the band gap (Eg) of the photocatalyst can be obtained from Kubelke–Munk equation $\left(\alpha \mathrm{hv}\right)=A{\left(\mathrm{hv}-\mathrm{Eg}\right)}^{n}$, where α is the absorption with respect to the optics coefficient and H is the energy with respect to photons. Since CeO_2_ is an immediate transition band gap, and UiO-66-NH_2_ is an indirect transition Eg, and the n value is 1/2 and 2 in this study. By plotting ${\left(\mathrm{hv}-\mathrm{Eg}\right)}^{2}$ and ${\left(\mathrm{hv}-\mathrm{Eg}\right)}^{1/2}$, the band gap can be determined by the tangent intercept. According to Figure 13a,c, the bandgap widths of CeO_2_ and UiO-66-NH_2_ are about 2.86 eV and 2.65 eV apart. Flat band potential (U_fb_) and semiconductor types were calculated using Mott–Schottky (MS) plots. According to Figure 13d,e, under the condition of 1 kHz, CeO_2_ and UiO-66-NH_2_ are fitted with positive slopes, both of which are the semiconductors type that belongs to the n type. Furthermore, the U_fb_ of CeO_2_ and UiO-66-NH_2_ are −0.63 eV and −0.64 eV, respectively. In general, U_fb_ has 0.1 eV more potential than a conduction band (CB) for most N-type semiconductors [38]. Therefore, the ratio of the bottom CB of CeO_2_ and the lowest unoccupied molecular orbital (LUMO) of UiO-66-NH_2_ to NHE is −0.73 eV and −0.74 eV, respectively. Combine the Eg value of VIOLET UV-vis DRS and $\mathrm{Eg}=E_{\mathrm{VB}}-E_{\mathrm{CB}}$. The HOMO ratio of CeO_2_ and UiO-66-NH_2_ to NHE is 2.13 eV and 1.91 eV, respectively.

### 3.7. Properties about Electrochemistry and the PL Emission Spectra

**Figure 14:**
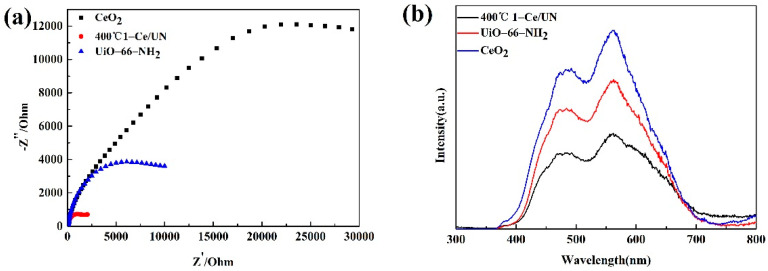
CeO_2_, UiO-66-NH_2_ and 400 °C 1-Ce/UN (**a**) EIS Nyquist diagram and (**b**) PL diagram.

The mechanism of photocatalytic activity was studied by EIS and the PL emission spectra emission spectroscopy, and the results are shown in Figure 14. EIS study is required to confirm and elucidate its good photocatalytic performance. From Figure 14a, it can be observed that 400 °C 1-Ce/UN has a smaller arc size than pure matter. Usually, according to EIS images, semi-arcs can reflect the recharge course because the size of it is related to the resistance. In addition, smaller arcs reflect smaller charge transfer resistance [39]. Therefore, EIS results show that the charge separation rate of 400 °C 1-Ce/UN is higher and the charge transfer rate is faster because of the lead-in of UiO-66-NH_2_. The results of EIS show that photocarrier separation of 400 °C 1-Ce/UN may be due to the formation of the heterojunction.

The photoluminescence (PL) emission spectrum further demonstrated the photoluminescence separation of electron–hole pairs at 400 °C 1-Ce/UN. According to Figure 14b, it can be found that the luminescence peaks of CeO_2_ and UiO-66-NH_2_ appear at about 470 nm and 570 nm, separately. The emission peak at about 470 nm may be a combination of electron hole radiation between CB and valence band (VB), and the emission peak at about 570 nm may be consistent with the good state emission [40]. In general, weak fluorescence intensity means a high photoinduced electron–hole pair separation rate, which can effectively restain the electron–hole for restructuring, and effective photocarrier separation can prolong the service life of photocatalyst, improve interfacial charge shift efficiency, and boost the photocatalytic efficiency [41]. The luminescence intensity of 400 °C 1-Ce/UN composite is obviously weaker than the pure substance, indicating that the electron and hole separation rate of 400 °C 1-Ce/UN can be effectively inhibited. The peak intensity of 400 °C 1-Ce/UN is lower than that of pure substances, which boosted the interfacial electronic shift and also corresponds to the consequences of photocatalytic degradation. Therefore, Ce/UN has a good photocatalytic property, which is consistent with the EIS conclusion mentioned above.

### 3.8. Photocatalytic Degradation Performance

**Figure 15:**
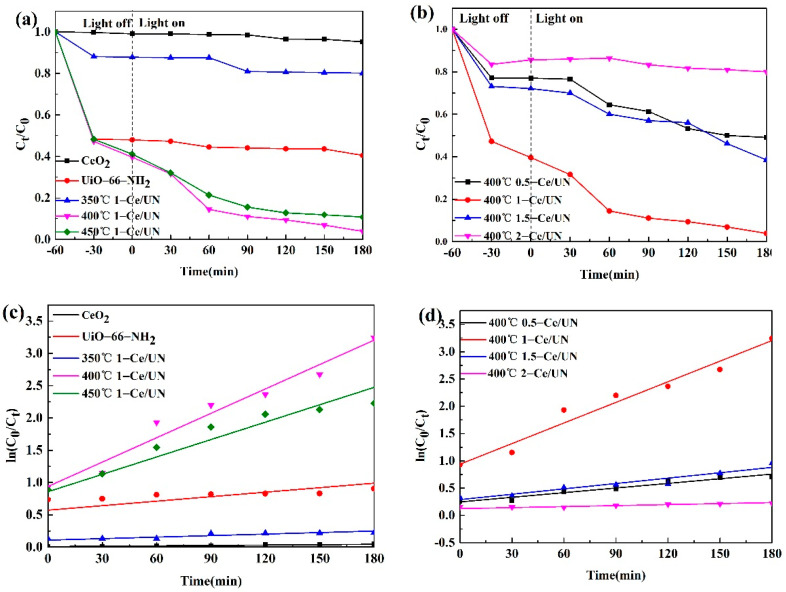
Degradation rate and first-order kinetic K diagram of RhB by CeO_2_, UiO-66-NH_2_, and Ce/UN: (**a**) degradation rate of different temperatures; (**b**) degradation rate of different proportions; (**c**) first-order kinetic K-value diagram of different temperatures; (**d**) first-order kinetic K-value diagram of different proportions.

The Photocatalytic property of 20 mg/L RhB was studied under simulated sunlight (room temperature) irradiation. The photocatalytic property of RhB was researched by comparing the photocatalytic property of RhB with the photocatalytic activity of RhB. The degradation rates of CeO_2_, UiO-66-NH_2_, Ce/UN at different temperatures, and Ce/UN at different proportions are studied. The results are shown in Figure 15. The catalyst is 10 mg in simulated wastewater. It can be seen from Figure 15a that the removal rates of RhB by CeO_2_ and UiO-66-NH_2_ alone reached 0% and 60% within 4 h, respectively. With the increase of CeO_2_ temperature, the photocatalytic properties of the sample increased first and then decreased. Thus, the temperature of CeO_2_ with the best degradation performance was found to be 400 °C. For pure CeO_2_, the adsorption efficiency was only 1% at the dark adsorption stage of 60 min. In addition, for UiO-66-NH_2_, the rate of adsorption is only 52% after dark adsorption for 60 min. The photocatalytic performance of 400 °C 1-Ce/UN composite in a dark adsorption stage is better than that of pure substances, and the adsorption efficiency reaches 60%. Because of the three-dimensional porous structure of UiO-66-NH_2_, the adsorption property of RhB is stronger. Thus, the rate of degradation of 400 °C 1-Ce/UN can reach 96%. The high degradation efficiency indicates that the combination of two-dimensional CeO_2_ flower nanosheets with UiO-66-NH_2_ has good oxidation capacity.

Compared with pure CeO_2_, the adsorption performance of Ce/UN is improved, so that the composite can effectively degrade pollutants. As shown in Figure 15b, the photocatalytic effect changes significantly after the two catalysts were combined, and with the increase of the CeO_2_ ratio, the photocatalyst properties of the composites increased first and then decreased. Thus, the photocatalytic efficiency can be improved. This phenomenon indicates that the structure of heterojunction is very favorable to photocatalytic degradation under certain conditions. The proper combination of UiO-66-NH_2_ is a vital reason to determine the photocatalytic property of UiO-66-NH_2_, and an excessive or insufficient reaction rate will decrease. It may be because of the obvious agglomeration of UiO-66-NH_2_ on the surface of CeO_2_ flower nanosheets. This may lead to accelerated charge recombination, which in turn inhibits carrier separation [42]. Meanwhile, the negative effects of excessive UiO-66-NH_2_ may be because of the shielding and blocking effects of active sites [43]. Therefore, the best photocatalytic activity of the composite when the suitable quality of UiO-66-NH_2_ is added_._

The aim is to further study the degradation process of RhB by a photocatalyst; the Langmuir-Hinshelwood (L-H) model $\text{-}\ln\left(\frac{C_{t\;}}{C_{0}}\right)=\mathrm{kt}$ was used to fit the reaction kinetics curve, in which K was the speed constant (min^−1^), C_0_ is the initial concentration of RhB (mol/L), and C_t_ is the concentration of RhB at time T (mol/L). According to Figure 15c, under the conditions of pure CeO_2_, UiO-66-NH_2_, 350 °C 1-Ce/UN, 400 °C 1-Ce/UN, and 450 °C 1-Ce/UN, the estimated rate constants K are 0.0002, 0.0023, 0.0008, 0.0126, and 0.0089, respectively. According to Figure 15d, under the conditions of 400 °C 0.5-Ce/UN, 400 °C 1-Ce/UN, 400 °C 1.5-Ce/UN and 400 °C 2-Ce/UN, the estimated rate constants k are 0.0028, 0.0126, 0.0033, and 0.0006, respectively. The results show that 400 °C 1-Ce/UN has a high rate constant, which is 20 times the pure CeO_2_ and 1.6 times the UiO-66-NH_2_, respectively. When CeO_2_ temperature is too high or the composite amount is too much, the photocatalytic activity of the composite material will be reduced, and the carrier in the composite material will be reduced. The low charge transfer rate and the accelerated reassociation of electron holes lead to the decrease of photocatalytic properties of the materials.

### 3.9. Photocatalytic Mechanism

**Figure 16:**
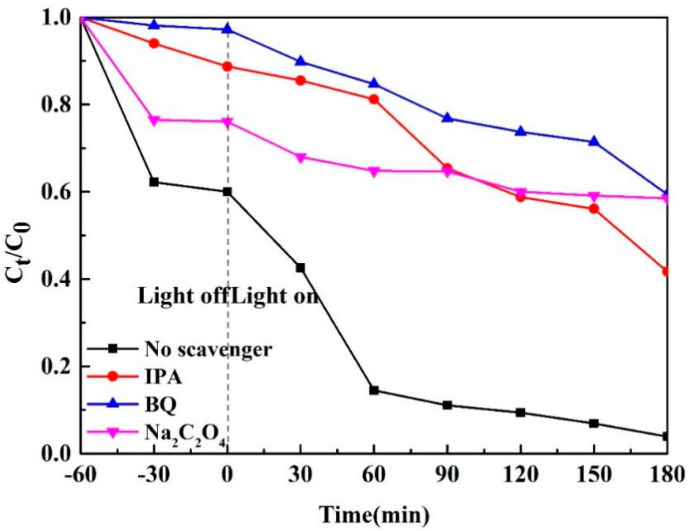
Effect of different scavenger on photocatalytic degradation of RhB by 400 °C 1−Ce/UN.

By introducing 0.1 mM free radicals of Na_2_C_2_O_4_ (holes^+^ scavenger)*,* isopropyl alcohol (IPA, •OH scavenger), and p-benzoquinone (BQ, $\cdot \;O_{2}^{-}$ scavenger), active material involved in the degradation related to photocatalysis was investigated, and the possible mechanism made was discussed, as shown in Figure 16. After 400 °C 1-Ce/UN, the degradation rate of RhB decreased to 96% within 4 h. After the addition of Na_2_C_2_O_4_, the photocatalytic degradation rate decreases, which can be concluded as (Equations (1)–(3)). The presence of Na_2_C_2_O_4_ significantly reduces the photocatalytic RhB degradation efficiency, indicating that the consumed holes are not conducive to the separation of e^−^−h^+^. With the BQ coming in, the degradation efficiency of RhB also obviously declined. Therefore, it can be seen that H^+^ and $\cdot \;O_{2}^{-}$ are the main species responsible for the degradation of RhB rather than •OH. In a degradation reaction, the •OH effect is relatively small:

(1) $${\mathrm{CeO}}_{2}/\mathrm{UiO}-66-{\mathrm{NH}}_{2}+\mathrm{hv}\to h^{+}+e^{-}$$

(2) $$e^{-}+O_{2}\to \cdot \;O_{2}^{-}$$

(3) $$O_{2}^{-}+h^{+}+\mathrm{RhB}\to \mathrm{Products}$$

**Figure 17:**
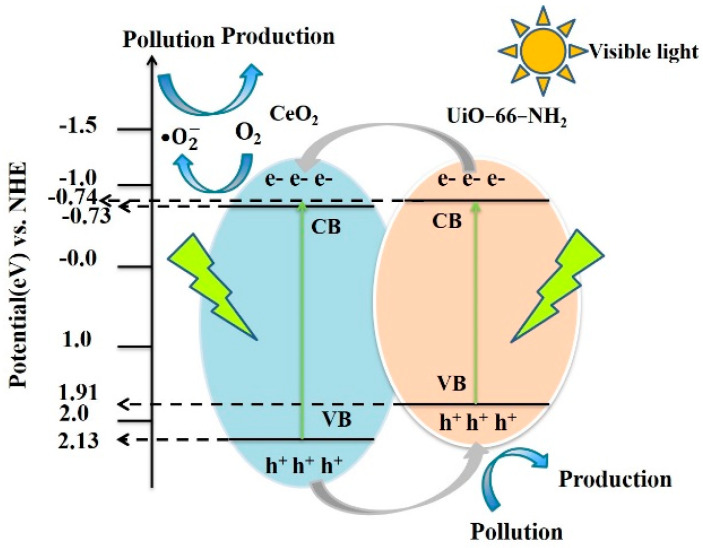
Photocatalytic mechanism of a Ce/UN composite.

According to the obtained results, the mechanism of Ce/UN composite made in photocatalysis was analyzed in depth (as shown in Figure 17). Both CeO_2_ and UiO-66-NH_2_ can generate e^−^−h^+^ upon visible light. Because of the potential difference between the CB of UiO-66-NH_2_ and CB of CeO_2_ at −0.74 eV, the light-generated electrons are transferred from the CB of CeO_2_ to CB of UiO-66-NH_2_. Both the LUMO of CeO_2_ and UiO-66-NH_2_ generate $O_{2}^{-}$ because their potential is higher than O_2_/$O_{2}^{-}$ (−0.33 eV vs. NHE) [44].

## 4. Conclusions

In this paper, a Ce/UN composite photocatalyst was in-situ synthesized by a hydrothermal method. The photocatalytic properties and degradation mechanism of composites were studied in detail. The results show that the Ce/UN composite photocatalyst shows better degradation efficiency of RhB than CeO_2_ and UiO-66-NH_2_ under the situation of simulated sunlight. The degradation rate of RhB reached about 96% after 4 h photocatalysis, which is much higher than CeO_2_ (4.5%) and UiO-66-NH_2_ (54%) under the same conditions. The enhancement of the photocatalytic performance of the Ce/UN may be because of the formation of heterojunction. It can be observed that Ce/UN composites present a 3D flower-like structure, and UiO-66-NH_2_ particles are covered on CeO_2_ nanosheets. This heterojunction structure may provide a large contact area for the transfer of e^−^−h^+^. In addition, a Ce/UN composite photocatalyst has lower charge transfer resistance and luminescence intensity compared with two pure substances according to the results of EIS and PL, which is beneficial to the separation of photocarriers and the improvement of the interfacial charge transfer efficiency. The free radical capture experiments show that H^+^ and $\cdot \;O_{2}^{-}$ are the main active species in the photocatalytic process.

## Figures and Tables

**Table 1:** The EDS results of the area “a” signed in Figure 9a.

Position	Composition (wt. %)				Possible Phases
	Ce	O	Zr	N	
a	76.2	24.5	3.23	0.91	CeO_2_, UiO-66-NH_2_

## Data Availability

The data presented in this study are available on request from the corresponding author.
